# Aquatic Toxic Analysis by Monitoring Fish Behavior Using Computer Vision: A Recent Progress

**DOI:** 10.1155/2018/2591924

**Published:** 2018-04-03

**Authors:** Chunlei Xia, Longwen Fu, Zuoyi Liu, Hui Liu, Lingxin Chen, Yuedan Liu

**Affiliations:** ^1^Yantai Institute of Coastal Zone Research, Chinese Academy of Sciences, Yantai 264003, China; ^2^The Key Laboratory of Water and Air Pollution Control of Guangdong Province, South China Institute of Environmental Sciences, MEP, Guangzhou 510065, China

## Abstract

Video tracking based biological early warning system achieved a great progress with advanced computer vision and machine learning methods. Ability of video tracking of multiple biological organisms has been largely improved in recent years. Video based behavioral monitoring has become a common tool for acquiring quantified behavioral data for aquatic risk assessment. Investigation of behavioral responses under chemical and environmental stress has been boosted by rapidly developed machine learning and artificial intelligence. In this paper, we introduce the fundamental of video tracking and present the pioneer works in precise tracking of a group of individuals in 2D and 3D space. Technical and practical issues suffered in video tracking are explained. Subsequently, the toxic analysis based on fish behavioral data is summarized. Frequently used computational methods and machine learning are explained with their applications in aquatic toxicity detection and abnormal pattern analysis. Finally, advantages of recent developed deep learning approach in toxic prediction are presented.

## 1. Introduction

Monitoring aquatic toxicology is a fundamental and essential task for risk assessment in aquatic ecosystems and water resource management. Sensing disturbance (e.g., pollutants or toxicants) in aquatic system is a crucial issue for early warning of water quality. Physicochemical parameters are the most widely adopted factors for examining disturbances to aquatic ecosystem. However, physicochemical sensors could only detect known toxicants within a period.

Behavioral monitoring is a type of biological monitoring that investigates the environmental conditions by examining the behavioral responses of indicator species and studies their relations to the surrounding environments. Behavioral monitoring is an efficient approach for long-term monitoring of the aquatic ecosystems and water quality assessment. Behavioral response of living biological organisms could represent the potential unknown risks in aquatic ecosystem. The accumulation effects of disturbance or chemical stress could be revealed by behavioral monitoring that is inexpensive comparing with physicochemical sensors [1, 2]. Fish and daphnia are the common indicator species for water quality assessment due to their sensitivity to the changes of environmental parameters [3–6].

In the early stage of behavioral monitoring of aquatic organisms, behavioral signal was represented by the strength of electrical fields in the surrounding water of the testing organisms. Electrodes were utilized to collect activity strength of fishes in a chamber [7]. Behavioral patterns are able to be characterized from the activity data. However, individual status is unable to be represented using the electrical signal.

In the middle of 1990s, computer vision is introduced to obtain quantitative behavioral data of fishes [8]. A number of video tracking systems have been developed for automatic observation of fish behaviors [9–12]. Fish behavioral monitoring has been carried out for tracking single individual and multiple individual in 2D and 3D space.

Video tracking based biological early warning system achieved a great progress in the last decade since advanced computer vision algorithms are developed and integrated to the behavioral monitoring system [13, 14]. Video tracking based biological monitoring becomes a common tool for obtaining behavioral data.

On the other side, computational models are widely studied for prediction of disturbance (e.g., toxicant) in aquatic ecosystem in the last decades. Rapidly developed machine learning techniques provide sophisticated approaches of detecting abnormal behaviors of aquatic organisms exposed chemical stress. Deep learning algorithms recently bring a strong influence to the artificial intelligence, including image understanding, speech recognition, and big data analysis. Recently, deep learning demonstrated a great potential to boost the toxicity prediction with behavioral responses. However, toxicity prediction using deep learning is still in the starting stage.

In this paper, we summarize the fundamental of video tracking and the development of behavioral monitoring. The state-of-the-art visual monitoring techniques in multiple individual tracking are stated and issues existing in the current monitoring systems are discussed. An overview of computational models and machine learning algorithm for investigating behavioral changes to toxic chemicals is presented and the recent research of toxicity prediction using deep learning is presented.

## 2. Video Tracking Based Behavioral Monitoring

### 2.1. Fundamentals

Monitoring individuals could reveal accurate and comprehensive behavioral changes to the surrounding environments. Individual behavior is more sensitive to toxic and pollution. Automatic individual behavioral monitoring using digital camera was proposed since middle 1990s [8]. Due to the physical limitation of computers and imaging techniques and hardware costs computer vision based behavioral monitoring was not extensively studied at that time. In the last decade, the blooming information technology promoted the development of biological monitoring. Computer vision based biological monitoring achieved a great attention from scientist and engineers. Numbers of behavioral observation systems were developed for monitoring individual behavior of aquatic organisms and behavioral sensing has been extensively studied for determining aquatic toxicity.

Images of testing organisms in the observation arena are acquired through a digital imaging sensor to investigate behavioral response of the observation targets. Individual organisms are usually detected and located from the observation scene by image processing algorithms and tracking algorithms accordingly link the individual locations in time series to generate movement trajectories of these individuals. Digital cameras are commonly utilized as the imaging facilities to record video clip or images of the observation area. To obtain real-time behavioral movement video clip should be recorded with frame rate of at least 30 fps.

Vision based behavioral monitoring includes video analysis and online real-time observation. Video analysis is considered as an offline monitoring technique. The procedure of behavioral movements of testing organisms is recorded in video clips in advance. The behavioral responses are examined on these recorded videos by using tracking and analysis software. This method could preserve the original video records for long periods but requires extreme large amount of storage units. Therefore, video analysis is not suitable for long-term behavioral monitoring. Video analysis was widely used at the beginning stage of vision based behavioral analysis, since insufficient computational ability of hardware costs much time to obtain the analysis results at that time. Recently, advanced computer technique promotes the development of online biological monitoring system in real application. Real-time behavioral observation is rapidly developed for immediate examining individual behavioral data from observation arena. Real-time behavioral monitoring could acquire individual behavioral data from live cameras in both laboratory or field conditions. Obtaining real-time behavioral data is a crucial issue for detecting environmental risks at early stage. Another advantage of real-time monitoring is that storing video clips is not necessary since behavioral data are quantified in the real-time process. Risk assessment could be directly calculated from numerical behavioral data by using statistical analysis or mathematical models. For long-term observation, these behavioral data do not need large amount of storage devices and they could be stored into text file or database for further analysis.

However, real-time monitoring system requires hardware with high performance and efficient recognition algorithms to process video stream from live camera data. Real-time image processing should satisfy the frame rate of 30 frames per second at least which indicates that biological individuals should be recognized and located from the images within 33 milliseconds per frame. This is another challenging issue for the sophisticated recognition and tracking algorithms achieving real-time performance.

### 2.2. Two-Dimensional Tracking

Vision based behavioral monitoring is categorized into 2D and 3D observation according to the dimension of behavioral data. Two-dimensional observation records the movement of biological individuals in a two-dimensional space which is actually observing the movement trajectories projected into a given plane. Commonly, a single camera is adopted to capture top view or side view images from the observation container. Recognition of individual and movement tracking are subsequently carried out on these captured 2D images.

At the initial stage, vision based behavioral monitoring tracks the two-dimensional movements of a single individual for behavioral analysis. Individual organisms, such as fish or daphnia, are recognized as a 2D point on the imaging plane. Movement trajectories are generated by connecting their 2D coordinates in temporal sequence. To improve the monitoring stability and to decrease the computational complexity, most behavioral monitoring systems work under controlled light condition. Simple image background and stable illumination are required to minimize the image noise from illumination variations. In two-dimensional single individual tracking, it is possible to obtain stable and accurate movement data with unsophisticated computer vision algorithms and low cost hardware. Biological individuals could be accurately extracted from the image background by only using thresholding or background subtraction.

Considering a digital image as a two-dimensional matrix, each image pixel is an element of the matrix. The pixel is valued in [0,255] in a gray image. The basic idea of thresholding is to divide image pixels into several groups. A function *f*(*x*, *y*) describes an image and (*x*, *y*) indicates the two-dimensional coordinate of a pixel in the image. *f*(*x*, *y*) represents the intensity value of a pixel located at (*x*, *y*) in a gray image. For a given threshold *T*, image segmentation could be represented by

(1) $$\begin{array}{c} f_{T}\left(\;x,y\;\right)=\left\{\begin{array}{cc} b_{0}, & f\left(x,y\right)<T\\ b_{1}, & f\left(x,y\right)\geq T. \end{array}\right. \end{array}$$

The segmentation results could be binarized when *b*_0_ = 0 and *b*_1_ = 225. Usually, images are transformed into binary images to extract individuals from background and analyzing their geometric feature. Thresholding is widely applied in object detection with less computational complicity.

Threshold segmentation includes global thresholding and local thresholding [15]. As explained above, global thresholding segments every pixel using the same threshold value for the entire image. A proper threshold value should be chosen for detecting individuals from the observation background according to the users' experience. Or global threshold could be automatically estimated according to the distribution of gray values of the whole image, for example, Otsu algorithm [16]. However, global thresholding has less stability against illumination noise. Local thresholding is proposed to improve the segmentation stability by calculating threshold for each pixel according to intensity distribution of its surrounding pixels, which is also known as adaptive thresholding. Adaptive thresholding is robust and stable to illumination noise and light diffusion and could effectively segment biological individuals in the observation arena.

Another commonly used individual detection method is background subtraction. In video sequence, image difference is obtained by the subtraction of current frame and predefined background image. Image difference is the subtraction of corresponding image pixels in two frames. In the subtracted images, background pixels present very small difference value since the static background shows less variation of intensity within a certain time period. While moving objects will be presented in large difference values, therefore, the observation targets could be effectively detected by applying a threshold to the difference image. The basic background subtraction is simple to implement but sensitive to illumination and color variations. Based on background subtraction, background modeling methods are proposed to improve the stability of detecting moving objects. In this method, background image should be continuously updated to minimize the effects of illumination noises. Background images are usually modeled using mean values or mixture of Gaussian models (MOG) [17].

Behavioral observation of two-dimensional single individuals has been widely applied to water quality monitoring and risk assessment in aquatic ecosystems.

However, behavioral data of single individual is not reliable and could not reflect actual status of test organisms. In aquatic toxicity monitoring, behavioral data of a group individual is necessary to obtain accurate water quality status and minimize false alarms. Behavioral observation of multiple individuals attracted great attention by biological and environmental scientists. Two-dimensional multiple individual tracking usually utilizes the same segmentation method (e.g., thresholding or image difference) for detecting target individuals. And movement trajectories of each individual are measured by multiple tracking algorithms. The most challenging issue suffered in multiple individual tracking is occlusion among individuals. Since interaction and aggregation frequently occurred among aquatic organisms (e.g., fish), accuracy of individual detection is severely reduced. On the other side, aquatic individuals present similar appearance (e.g., color, texture), body shape, and size. It is rather difficult to correctly identify individuals from each other only by using appearance features.

**Figure 1:**
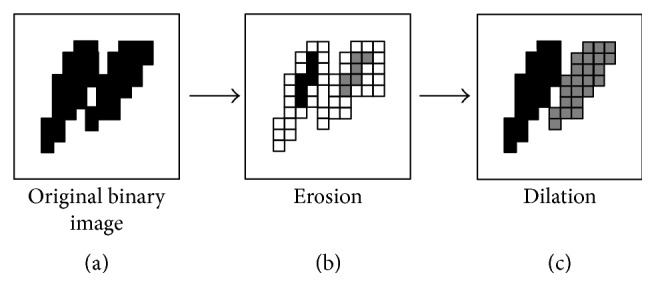
Separating attached fish images using morphological operations [19].

**Figure 2:**
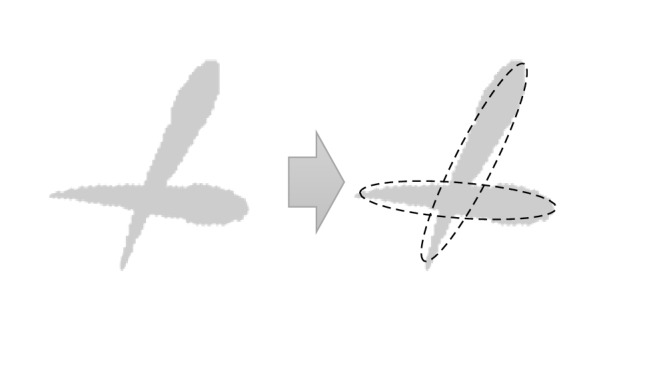
Fitting individual fishes from occlusions.

As the development of computer vision, numerous individual recognition schemes are reported to track individual movement from occlusions. Prediction models, such as Kalman filter or particle filter, are proposed to predict the movement trajectory to follow the individual in occlusion [18]. Image processing algorithms are utilized to separate the connected individual images. Erosion-dilatation is a common morphological operation to separate the connected individual fishes [19]. The erosion-dilatation process is illustrated in Figure 1. A binary image of two attached fishes is obtained in Figure 1(a). The binary image is divided into two small blobs after erosion (Figure 1(b)). The dilatation operation is followed to recover the separated blobs to its original shape. The number of erosion and dilatation operations should be decided according to the occluded area. Erosion-dilatation is effective in separating the slightly attached individuals and computational inexpensive. However, morphological operations are not suitable to heavy occlusion, such as cross overlapping of two fishes (Figure 2).

Contour fitting is introduced to find individuals from heavy occlusions. In fish behavioral monitoring, an ellipse model is adopted to detect individual fishes since fish contour is close to an ellipse. Individual fish could be accurately detected by fitting the ellipse equation using least squares method. This method is effective in finding individual fishes from cross overlapping (Figure 2).

**Figure 3:**
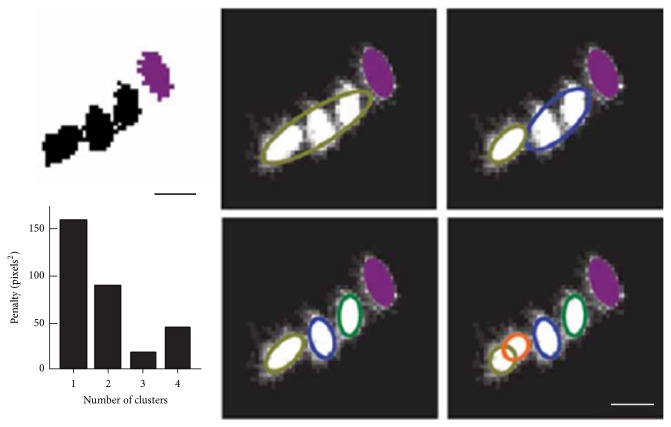
Procedure of extracting individuals from occlusions using clustering and ellipse fitting [20].

Recently, tracking software, Ctrax, showed stable tracking ability of multiple individuals [20]. In Ctrax, individual images are extracted using background subtraction. A constant velocity prediction model is presented to predict the movement trajectories and select the optimal matching movement trajectories. Individuals are identified from occlusions based on ellipse fitting and pixel clustering. Ellipse fitting is firstly calculated to determine whether the given image contains multiple individuals (Figure 3). High fitness indicates that the image is a single individual and further segmentation is not required. Otherwise, image pixels are grouped into several clusters according to the Euclidean distance. Subsequently, ellipse fits are calculated on each cluster and a penalty value is given to represent the overall fitness of each cluster. Different numbers of clusters are tested to determine the number of individuals in the image. Number of individuals is determined by choosing the cluster with least penalty.

**Figure 4:**
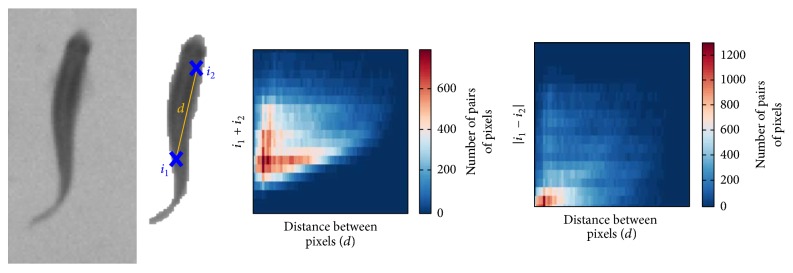
Calculation of “Fingerprint” features [21].

In multiple biological individual tracking, loss of tracking targets and following incorrect targets are the most challenging difficulty due to occlusions among individuals. Movement trajectories of individuals could be swapped when the tracking algorithm detects incorrect targets after occlusion. The tracking error will be increased when observing a large number of individuals. Recently, a “Fingerprint” algorithm is developed to identify individuals from each other [21]. The “Fingerprint” describes a unique image feature for each individual. The idTracker behavioral tracking software is developed based on the “Fingerprint” technique and successfully applied to record group behavior of fish. “Fingerprint” is obtained by calculating the distance and intensity of arbitrary two pixels in the individual image (Figure 4). Define *d* as a Euclidean distance of arbitrary two points *i*_1_ and *i*_2_ in the individual image. A three-dimensional vector (*d*, *i*_1_, *i*_2_) is obtained from all combinations of pixel pairs of the individual image. An intensity map is obtained by calculating 2D histogram of the intensity sum of every pair of pixels (*i*_1_ + *i*_2_). Similarly, a contrast map represents the 2D histogram of (|*i*_1_ − *i*_2_|). The proposed tracking system produces a reference “Fingerprint” feature for each individual at the beginning of tracking. In the following frames, tracking algorithm identifies individuals by comparing every individual image with the reference “Fingerprint” feature. The best matching individuals are chosen to produce the tracking trajectories. The “Fingerprint” feature is translational and rotational invariant since only Euclidean distances are calculated and robust to posture changes. This method achieved excellent accuracy of tracking multiple individuals after occlusion. Calculating the “Fingerprint” feature is rather computationally expensive which requires high performance computers to extract behavioral data from video clips. It is not possible in real-time monitoring yet.

**Figure 5:**
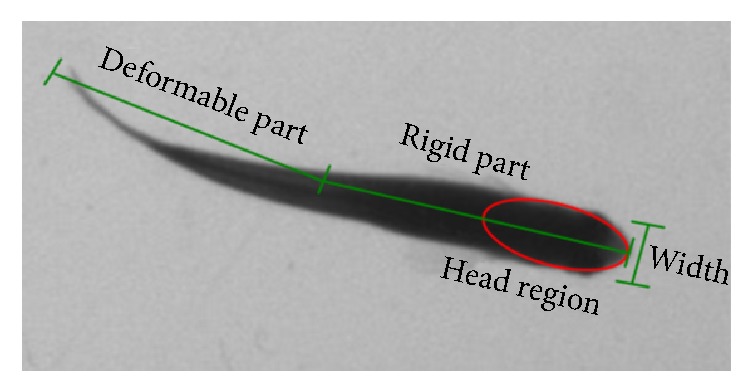
Fish tracking by detecting fish head region [23].

Individual fishes are commonly treated as a single blob and centroid of the blob is utilized to generate the movement track in behavioral monitoring system for early warning of toxicity. However, detailed body motion of individuals could not be explored with only movement tracks. Sophisticated algorithms are reported to examine body shapes during observation. Posture information contains more precise behavioral response regarding specific stimulation and environmental changes. An automatic fish posture measurement is presented by utilizing image identification of fish head and tail [22]. Identification of fish head and tail is developed by examining the gray level appearance features. Subsequently, the fish body is represented by three points: fish head, centroid, and fish tail. And a simplified fish posture is described by an angle formed by the head and centroid of fish. This posture description also represents the movement direction of fish. Directional changes and self-turning of fishes during observation could be investigated that might be useful parameters for examining toxicity. This method is effective in low resolution video and with low cost hardware. The water depth is limited since tracking from occlusions could not be solved. A study of multiple fish tracking from occlusions is presented by detecting fish head from the overall observation image [23]. In this work, a fish body is assumed as rigid part and deformable part (Figure 5) since fish head is rigid and has less variation in appearance. Extremum detection and ellipse fitting are utilized to locate fish head region in the observation frame. The Kalman filter is applied to track fish head region. Discontinued movement trajectories caused by occlusions are linked according to time and distance. Thus, a complete movement trajectory of fish is generated from occlusions.

A statistical deformable model is proposed to track the two-dimensional fish body [24]. One of the advantages of deformable model is to fit partially occluded objects that indicate that fish could be located from occluded fish images with deformable model. The variations of fish body are modeled by active shape model which is robust to shape changes in the complicated background [25]. The shape of fish body is described by a vector *x*_*i*_ with *n* landmarks:

(2) xi=xi,0,yi,0,xi,1,yi,1,…,xi,n−1,yi,n−1T,i=1,…,N,

where (*x*_*i*,*j*_, *y*_*i*,*j*_) are the coordinates of the *j*th landmark of the *i*th shape in the training sets, *N* is the number of images in the training set, and *T* is the transpose operator. After training, fish body could be represented approximately by the mean shape ($\overset{-}{x}$) and weighted modes of variations:

(3) $$\begin{array}{c} x_{i}=\overset{-}{x}+Pb, \end{array}$$

where **P** is the matrix of eigenvectors and **b** is the vector for weights. The deformable fish model changes its shape to fit a given fish image by tuning the weight vector **b**.

**Figure 6:**
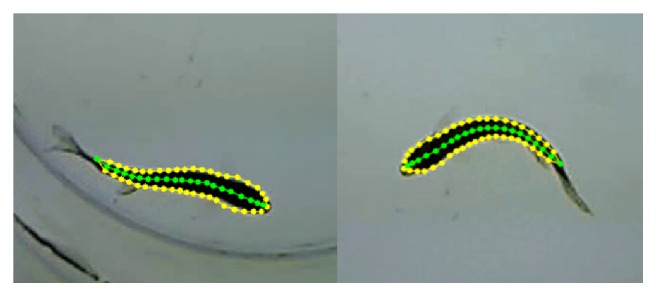
Individual fishes represented by deformable models (yellow contour) and their skeletons (green line) [24].

Figure 6 illustrates fish measurement results by the proposed deformable fish model showing the flexibility of outstanding fitting to the actual fish image. The skeleton of fish is described by a series of points calculated from the symmetric pairs of landmarks on the fish contour. In this work, fish skeleton contained 20 points that provides a high dimensional pose vector for analysis of complex behavior.

Beside deformable model, a chain of rectangles is presented to model a fish body for behavioral tracking [26]. Fish nose is located by examining the point maximum curvature along fish boundary.

**Figure 7:**
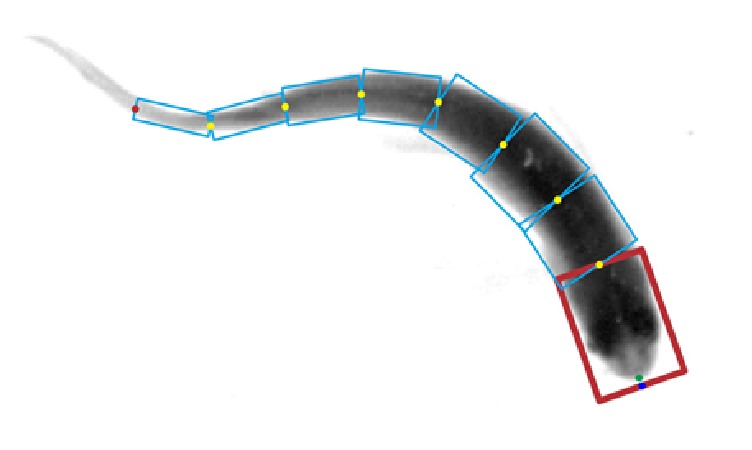
A fish represented by a chain of rectangles [26].

A group of rectangles rec_*i*_  (*i* = 1,…, *n*) are defined where *n* indicates the total number of rectangles. The adjacent rectangles are jointed at the midpoint on front edge as presented in Figure 7. After locating fish head and calculating its orientation, fish pose is estimated based on rectangle chain fitting. Since no prior knowledge of fish shape is involved, the fitting process searches for all possible configurations in random direction. The rectangle covers the largest area of fish image which is considered as an optimal fit. This method has been tested on 20 individual fish group.

### 2.3. Three-Dimensional Tracking

**Figure 8:**
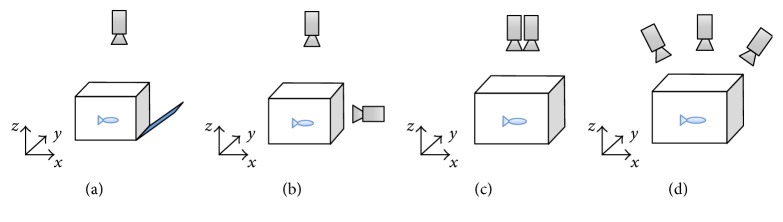
Structures of 3D observation systems.

Many of the indicator species (e.g., fish) in aquatic monitoring are active in three-dimensional space; however, 2D behavioral tracking will loss the observation accuracy and is not able to record comprehensive movement status of the individuals. For example, top view 2D fish observation cannot measure the depth of a fish. Therefore, three-dimensional behavioral monitoring is necessary to investigate the detailed individual movement. Three-dimensional behavioral tracking is studied for decades and achieved great progress in recent years. Various structures of 3D observation devices are developed. The 3D observation facilities are summarized in Figure 8, including single camera with auxiliary facilities, top-side view cameras, binocular cameras, and multiple-view cameras.

Single camera 3D observation system is usually constructed with mirrors (Figure 8(a)). The camera captures biological individuals from observation arena and its mirrored images simultaneously. A target individual is detected in the observation area and paired with its projected image on the mirror. Its 3D coordinates are obtained according to the projection relations between observation container and the mirror. Observation using single camera could reduce the cost of hardware but the mirror should be installed with accurate calibration. The mirror should be attached to one side of the container with a certain angle; thus, the projected images on the mirror might be distorted. The tracking accuracy will be highly decreased when increasing the number of target individuals.

Top-side view structure consists of two cameras capturing images from top and side views of the container, respectively (Figure 8(b)). Top view camera measures individual movement projected into *x*-*y* plane, while side view camera observes individuals from *y*-*z* plane. Thus, 3D coordinate of the individual could be calculated by combining positions from top and side views. The measurement accuracy is relatively low in this kind of structure.

**Figure 9:**
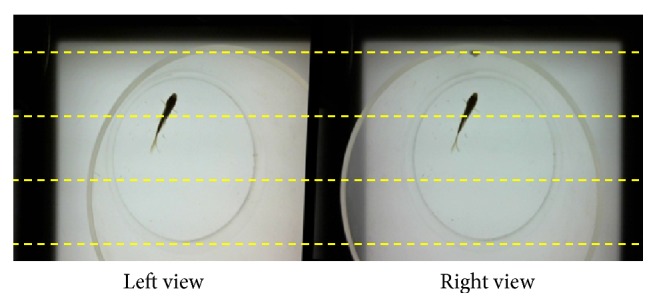
Rectified views of stereo camera.

Binocular camera structure is a typical structure of stereo vision. The two cameras should be placed on the same plane and their optical axis should be in parallel. Stereo cameras should be calibrated to obtain intrinsic parameters of cameras and rectify the optical distortion of lens. An example of rectified stereo images is presented in Figure 9. Each point of the objects projected into left and right view is aligned along the epipolar line. Thus, the corresponding points could be paired for 3D reconstruction. In 3D fish tracking, individual images are detected from left and right view, and its 3D coordinate is calculated based on the epipolar geometry [27].

Multiple-view cameras are the most complicated structure in 3D observation. More than three cameras are designed to capture the tracking targets concurrently. This kind of structure could be applied to tracking a large number of individuals or in large scale space [28, 29].

## 3. Toxic Analysis

The water quality monitoring technique on the basis of behavior changes of aquatic organisms is an advanced technique in the field of monitoring water environment change. It possesses the characteristics such as high stability and reliability, easy maintenance, and low operating cost.

The change of behavioral strength of aquatic organism has a regular pattern when they are stimulated by external factors such as contaminations: the change of behavioral strength correlates with exposure time under a certain pollutant concentration or correlates with pollutant concentration under a certain exposure time. The behavioral indicator used for monitoring and assessing water quality mainly includes escape behavior (e.g., floating, rapidly swimming, and circuitous frequency), motor behavior (e.g., velocity, height, swerve frequency, swing frequency, dispersion, and social event), and breathing behavior (breathing rate, depth of respiration). The purpose of analyzing behavioral data is to evaluate the pollution status in aquatic systems and potential risk assessment by identifying abnormal behavioral patterns from the monitoring data.

Quantitative analysis and processing of original behavioral data are necessary to detect the water pollution status, since the original behavioral data is movement track of aquatic organisms which could not explain mechanisms, phenomenon, or predicting toxicity. In the phase of analyzing and processing data, firstly, we preliminarily conjecture about pollutant concentration according to the synthesis of toxicological factors and analysis of aquatic organism behavior change pattern. Secondly we establish relation model between individual organisms and environment. Then, based on the more sensitive bioelectricity signal, a more accurate behavior data and reliable toxicological information can be obtained that the accuracy of pollutant concentration and characteristic of pollutant is increased. At last, the method of reducing interference from environment and individual organism is also necessary.

### 3.1. Computational Analysis

A number of computational and mathematical models are extensively studied to characterize the behavioral patterns and to detect abnormal behavioral changes under stimulates of toxicity. The original behavioral data is usually collected as individual movement trajectory. Various movement parameters could be calculated from the movement data, such as velocity, angular speed, acceleration, and meander [30]. Movement trajectories and movement parameters could be considered as behavioral signals. Therefore, many signal processing approaches are applied to investigate behavioral responses of aquatic organisms exposed to toxic chemicals [13].

#### 3.1.1. Fourier Transform

Fourier transform is a classical approach in signal processing that can decompose a time series signal into frequencies and to represent the features in frequency domain. Fourier transform is introduced to characterize behavioral pattern by transforming the movement tracks from spatial domain to frequency domain. Park et al. reported a study of characterizing behavioral changes of Medaka fish before and after treatment by diazinon using 2D fast Fourier transform (FFT) [2]. The fishes treated with diazinon showed more shaking and irregular turnings comparing with their normal behavior. These abnormal movements were featured in frequency domain by 2D FFT. The amplitude of 2D FFT presented clear different patterns before and after treatment. 2D FFT was efficient in describing movement data compared to the movement parameters (e.g., speed and meander). Another advantage of Fourier transform is that observation noise could be effectively removed by transforming movement trajectories from time domain to frequency domain.

#### 3.1.2. Wavelet Transform

Wavelet transform has the advantage of processing information from nonstationary signals and could extract multiresolution spatial-temporal features. Wavelet transform is adopted to analyze the individual movement trajectories [31]. A discrete wavelet transform is presented to select optimal features from behavioral data [32]. Behavioral variables with high value of wavelet coefficients were chosen as the representative features, that is, meander, angular acceleration stop duration, maximum distance, and the number of backward movements. Additionally, wavelet analysis was effective in variable selection regarding detecting movement patterns.

#### 3.1.3. Fractal Analysis

Fractal analysis presents fractal characteristics of movement trajectories and could be used to examine the movement patterns of observed individuals. The distribution pattern of movement tracks was described by calculating their fractal dimension using box counting method [33, 34], since movement tracks showed very different spatial distribution before and after treatment. For example, movement tracks were uniformly distributed in normal conditions. And after treatment, movement might happen at nearly the boundary of arena or aggregated in a certain area. Accordingly, these movement patterns could be represented by calculating the fractal dimension of the movement tracks. Fractal dimensions are effective in examining abnormal changes of behavioral patterns under chemical stress.

#### 3.1.4. Permutation Entropy

Permutation entropy measures the complexity of natural time series signals [35]. Permutation entropy was successfully applied to characterize the time series behavioral data and detecting abnormal behavioral responses of Medaka fish and* Drosophila* under chemical stress [36, 37]. Permutation entropy could effectively detect the behavioral changes of target individuals under chemical stress. Permutation entropy increased after the chemical treatments since movement speed of individuals decreased after the treatments [37].

### 3.2. Machine Learning

Machine learning is evolved from pattern recognition and computational learning that can learn patterns from given data. Machine learning is mainly categorized into supervised learning and unsupervised learning. Supervised machine learning consists of training and prediction steps. Patterns are learnt from the labeled training data. Once a machine learning model is trained, it could perform predictions from the new input data. With supervised learning, abnormal behavioral changes are possibly detected by a pretrained classification model. Unsupervised machine learning is to find the hidden structure from unlabeled data. One widely used unsupervised learning machine is called clustering. The unlabeled data are categorized into several groups according to certain metrics. Clustering reveals the difference among groups of the given data. Clustering is often utilized to summarize movement patterns of biological individuals.

Analyzing movement tracks by machine learning methods is usually combined with movement parameters and feature extraction methods to achieve optimal analysis accuracy and reducing computational costs. For example, some basic movement parameters (e.g., speed, acceleration, meander, and stop duration) or numerical analysis functions (e.g., fractal dimension) are obtained as the input data to train machine learning models.

#### 3.2.1. Multilayer Perceptron

Multilayer perceptron (MLP) is a typical structure of artificial neural network and widely applied to pattern classification and prediction tasks in many fields, for example, image understanding and speech recognition. MLP contains 3 layers usually: input layer, hidden layer, and output layer. Each layer is composed of neuron nodes which are the activation functions mapping the weighted inputs from input layer to output layer. Layers are connected by full connection of nodes with weights. In the training process, input variables are delivered from input to output layer though nodes in each layer. Output errors are feedback to the input layer and weights are adjusted to minimize the output error; this is the procedure of backpropagation. Previous studies reported detecting behavioral response of aquatic organism exposed to toxic water using MLP [30, 38]. Kwak et al. utilized MLP to investigate the movement pattern of medaka fish treated by insecticide [30]. In this work, a smooth movement pattern was observed as normal behavior, while a shaking movement pattern was found after treatment by diazinon. Parameters characterizing the movement tracks, such as speed, degree of backward movements, stop duration, turning rate, meander, and maximum distance within 1 minute, were selected to construct the MLP classifier. Artificial neural network demonstrated the reliability for accurately detecting abnormal behavioral pattern under toxic chemical exposure.

#### 3.2.2. Self-Organizing Map

A self-organizing map (SOM) is an unsupervised neural network that reveals the similarity of input data. Input data are automatically clustered according to the distance between weights and input values. The SOM is efficient to visualize multidimensional and complex data in an intuitive low dimensional plot [39]. SOM became one of the popular models in ecology studies [40, 41]. Quantifying behavioral patterns using SOM are extensively studied for examining abnormal behavioral responses under chemical stress or bacterial infection [42–44]. For example, Liu et al. extracted several movement parameters (including speed, average speed, and locomotory rate) from observation data of zebra fish for investigating behavioral changes treated by formaldehyde [43]. The parameters were calculated from movement sequences with 0.75 s time interval. Six typical movement patterns were classified by the SOM. The accuracy of examining behavioral responses before and after treatment was in an acceptable range (70.1–81.2%).

#### 3.2.3. Hidden Markov Hodel

A hidden Markov model (HMM) is defined based on Markov model by introducing hidden states to Markov process. HMM could infer optimal hidden states of individuals from the observed behavioral sequences. HMM could explain behavioral data in depth comparing with conventional time sequence analysis. HMM was successfully applied to exploring behavioral pattern for zebrafish and* Daphnia* with chemical treatment [44, 45]. According to the shape of movement segments, behavioral patterns are defined and considered as states of HMM. These behavioral states were extracted from the movement tracks; subsequently, transition probability matrix (TPM) and emission probability matrix (EPM) were obtained for estimating optimal state sequences. Behavioral changes of zebra fish exposed to formaldehyde were studied by using HMM [45]. After training, TPM and EPM were learnt from observed data. TPM and EPM presented the difference before and after chemical treatment. Accordingly, movement tracks were successfully constructed by the HMM. The reconstructed movement tracks were close to real data.

#### 3.2.4. Deep Learning

Deep learning (DL) is currently the most advanced algorithm in machine learning. It is evolved from the artificial neural network. Deep learning achieved remarkable success in image understanding, speech recognition, and big data analysis [46]. Deep learning has become the cutting-edge approach in artificial intelligence field with advanced supercomputers and big data. Deep learning is developed based on architectures of artificial neural networks with large number of hidden layers and millions of parameters to enhance the capability of learning.

Deep learning has not been widely studied in environmental science. In particular, deep learning has not been introduced to toxicity assessment in aquatic ecosystems. Recent studies prove that DL has a great potential achieving superior performance of prediction in computational toxicity [47]. DL outperformed many other state-of-the-art machine learning algorithms (e.g., support vector machines and random forests) in predicting toxic effects of chemical compounds.

One of the advantages is that deep learning algorithms can automatically learn features from the input data. Selecting optimized feature description is an essential task for the conversional machine learning methods (e.g., neural network and support vector machines). Many feature extraction algorithms (e.g., PCA) are reported for obtaining features to achieve optimized learning accuracy from the environmental data [48]. Improper feature representation could highly decrease the performance of machine learning methods. Deep learning could learn highly effective feature representations from training data and can improve the performance even with few training examples. Deep learning in toxicity prediction is still in its initial stage. Developing deep learning based toxicity analysis and prediction software is an urgent demand for biological and environmental scientists. We believe deep learning could bring a great impact on environmental sciences including biological early warning for aquatic toxicity assessment.

## 4. Conclusions

Behavioral sensing plays an important role in risk assessment in aquatic ecosystems. Precise individual behaviors and body states are possible to be measured by advanced computer vision algorithms. Frequently occurred occlusion is still the main obstacle in multiple individuals. Sophisticated algorithms are developed to extract detailed body feature to improve the tracking accuracy of large amount of individuals.

Abundant detailed behavioral data are produced for toxicity prediction by the current behavioral monitoring systems. Pattern analysis of aquatic organisms has been carried out against various stimuli from toxic chemicals. Numbers of computational methods explored behavioral patterns. Rapidly developed machine learning algorithms are superior in predicting aquatic toxicity. Although the revolutionary deep learning has not been widely adopted in environmental sciences, it shows a great potential to achieve remarkable results in behavioral analysis. We expect that more and more behavioral analysis tools are developed based on deep learning for environmental monitoring and ecological studies.

However, video tracking based toxicity sensing is still in its initial stage. Many issues should be overcome to improve the toxicity sensing methods for reliable real applications. For example, the recent tracking schemes performed remarkable ability in tracking a group of individuals from occlusions. But these methods are rather computationally expensive that could produce real-time data for online monitoring at the current stage. For behavioral based toxicity analysis, standard criteria for quantifying behavioral activity should be developed. And 3D behavioral data analysis has not been widely conducted that could provide more detailed behavioral patterns. More studies need to improve the behavioral based aquatic monitoring and to explore relation between behaviors of aquatic organisms and the surrounding environments.
